# Cysteine [2,4] Disulfide Bond as a New Modifiable Site of α-Conotoxin TxIB

**DOI:** 10.3390/md19020119

**Published:** 2021-02-22

**Authors:** Baojian Zhang, Maomao Ren, Yang Xiong, Haonan Li, Yong Wu, Ying Fu, Dongting Zhangsun, Shuai Dong, Sulan Luo

**Affiliations:** 1 Key Laboratory of Tropical Biological Resources of Ministry of Education, Key Laboratory for Marine Drugs of Haikou, School of Life and Pharmaceutical Sciences, Hainan University, Haikou 570228, China; zhangbaojian96@163.com (B.Z.); syphurmm@163.com (M.R.); ncdxxy@163.com (Y.X.); lihaonan_suger@163.com (H.L.); fuying926@163.com (Y.F.); zhangsundt@163.com (D.Z.); 2 Medical School, Guangxi University, Nanning 530004, China; wys211@163.com

**Keywords:** α-Conotoxin TxIB, peptidomimetics, disulfide bond, high yield, activity

## Abstract

α-Conotoxin TxIB, a selective antagonist of α6/α3β2β3 nicotinic acetylcholine receptor, could be a potential therapeutic agent for addiction and Parkinson’s disease. As a peptide with a complex pharmacophoric conformation, it is important and difficult to find a modifiable site which can be modified effectively and efficiently without activity loss. In this study, three xylene scaffolds were individually reacted with one pair of the cysteine residues ([1,3] or [2,4]), and iodine oxidation was used to form a disulfide bond between the other pair. Overall, six analogs were synthesized with moderate isolated yields from 55% to 65%, which is four times higher than the traditional two-step oxidation with orthogonal protection on cysteines. The cysteine [2,4] modified analogs, with higher stability in human serum than native TxIB, showed obvious inhibitory effect and selectivity on α6/α3β2β3 nicotinic acetylcholine receptors (nAChRs), which was 100 times more than the cysteine [1,3] modified ones. This result demonstrated that the cysteine [2,4] disulfide bond is a new modifiable site of TxIB, and further modification can be a simple and feasible strategy for the exploitation and utilization of α-Conotoxin TxIB in drug discovery.

## 1. Introduction

Nicotinic acetylcholine receptors (nAChRs) are one kind of pentameric ligand-gated ion channels that are widely expressed in the central and peripheral nervous systems. Several nAChRs are involved in pathologies and disorders, and the ligands of these receptors may exhibit therapeutic potential [1]. α6-Containing nAChR subtypes are prominently expressed in dopaminergic neurons of the ventral tegmental area and the substantia nigra pars compacta, making them potential targets for Parkinson’s disease, nicotine addiction and alcohol addiction [2,3,4,5]. 

α-Conotoxins, which act as competitive nAChRs antagonists, are the most widely studied conotoxins. Preliminary studies have found various α-Conotoxins with good activity and specificity for nAChR subtypes [6]. They have good guiding significance for studying the function and structure of nAChRs and the associated diseases. For example, VnIB is a specific ligand for human α6/α3β4 receptor with an IC_50_ of 5.3 nM [7], [S9K]TxID has a specificity for mouse α3β4 nAChR [8] and GeXIVA can effectively inhibit α9α10 nAChR [9]. Recent research has shown that ImI-modified paclitaxel polymeric micelles can target non-small cell lung cancer tumor cells which overexpressed α7 nAChR [10]. α-Conotoxins are cysteine-rich peptides, with disulfide bonds to form the accurate pharmacophoric conformation. Disulfide bonds are unstable under reducing conditions, so the strategy of disulfide bond engineering has potential value [11,12,13,14,15,16,17,18,19,20,21,22]. Presently, there are a variety of strategies employed in conotoxin disulfide bond modification, such as disulfide bond replacement by thioether, lactam bond, selenoether, selenylsulfide, 1,2,3-triazole, diselenide and dicarba [23,24]. Disulfide bond replacement by 1,2,3-triazole was studied in conotoxin MrIA and GI. Astrid Knuhtsen et al. found that replacement of the disulfide bond with a triazole group maintained its biological activity, and stability tests in rat plasma indicated that it significantly improved its plasma half-life by 10-fold compared with native GI [25,26].

α-Conotoxin TxIB is the most selective antagonist of rα6/α3β2β3 nAChR with an IC_50_ of 28.4 nM [27]. It has great therapeutic value for nicotine and alcohol addiction. You et al. found that the globular TxIB is an excellent antiaddiction drug on nicotine-induced conditioned place preference (NIC-induced CPP) [28]. However, as a polypeptide with two disulfide bonds, TxIB suffers from low synthetic yield, poor metabolic stability and low bioavailability in vivo, thus affecting its pharmaceutical potential. In order to prepare TxIB efficiently, Wu et al. explored the free oxidation of TxIB to get only the globular isomer, but the isolated yield was not reported [29]. Yu et al. explored the use of *Escherichia coli* expression to generate TxIB and get about 3 mg/L r-TxIB (GCCSDPPCRNKHPDLCM) with an additional Met residue at its C-terminus, which still showed good selectivity to inhibit rat α6/α3β2β3 nAChR [30]. To increase stability, Li et al. used a normal GGAAGAG linker to cyclize TxIB and improve its serum stability [31]. While some of the amino acid residues of natural α-Conotoxin TxIB have a reactive side chain, like Ser, Asp, Arg, Asn, Lys and His, these reactive side chains make it a challenge for selective cyclisation and modification. The yield for formation of two disulfide bonds by orthogonal protection strategy is normally around 15% and backbone cyclization is even less, which increases the difficulty and costs substantially during research and development. 

**Figure 1:**
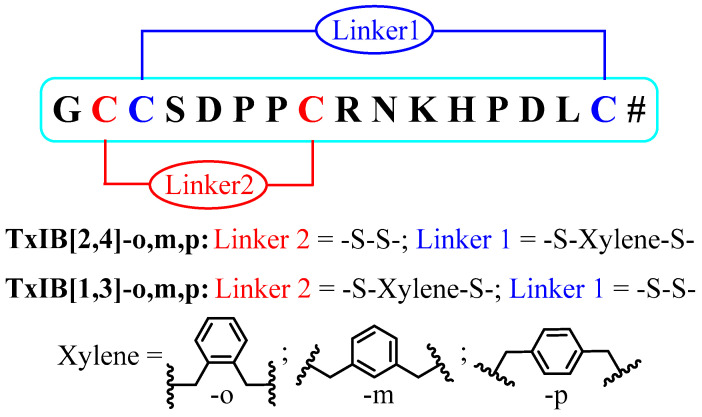
Six α-Conotoxin TxIB analogs (# signify C-term amidation).

Xylene dibromides are highly reactive linkers used in peptide cyclization [32]. In this experiment, three xylene dibromide scaffolds (m-, o- and p-xylene dibromide) were individually reacted with one pair of the cysteine residues ([1,3] or [2,4]), and iodine oxidation was used to form a disulfide bond between the other pair (Figure 1). This increased the yield significantly and enhanced the serum stability slightly. TxIB[2,4]-o, m, p analogs still show obvious inhibition on α6/α3β2β3 nAChR, which is 100 times more than the TxIB[1,3]-m, o, p analogs. This will allow guidance for further structure modification and will be conducive to the applicability of TxIB as a stable, bioavailable probe or drug that is specific for α6/α3β2β3 nAChR.

## 2. Results

### 2.1. Reaction Condition Optimization and Synthesis of TxIB Analogs

The resin-attached peptide was synthesized by standard Fmoc solid-phase synthesis technology, then cleaved and purified by preparative HPLC. The purity of each linear peptide was above 90%. The first and the third cysteine residues of linear peptide TxIB[2,4]-L were protected by acetamidomethyl (Acm), and the second and forth cysteine residues of TxIB[1,3]-L were protected by Acm. The molecular weight of TxIB[1,3]-L and TxIB[2,4]-L detected by UPLC-MS were 1886.14 and 1886.36 Da, which is consistent with the theoretical molecular weight 1886.17 Da (Supplementary Material Figure S1). The products were lyophilized for the next step.

**Figure 2:**
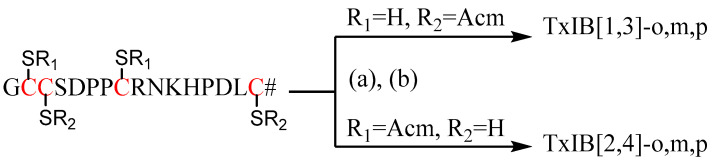
Reagents and conditions. (a) Xylene dibromide, NH_4_HCO_3_, ACN-H_2_O, N_2_, rt. (b) I_2_, TFA, ACN-H_2_O, N_2_, rt (# signify C-term amidation).

**Figure 3:**
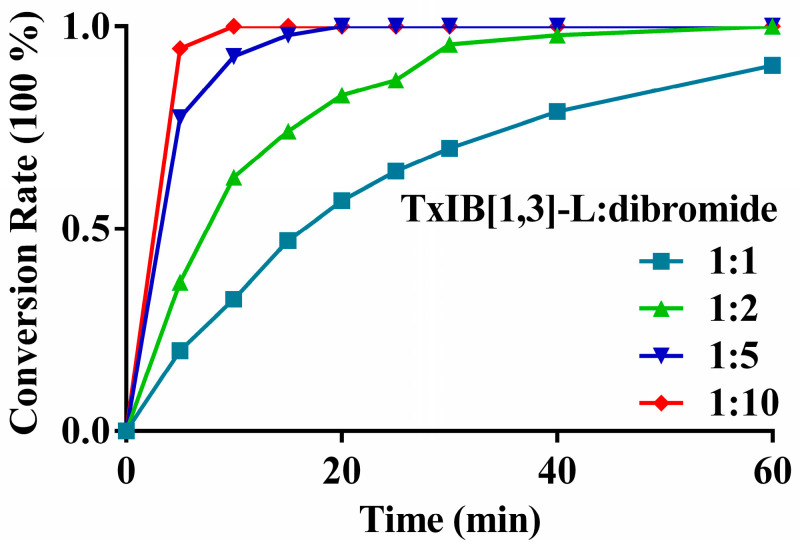
The conversion rate of TxIB[1,3]-L reacted with different ratios of p-xylene dibromide.

The final products were synthesized in two steps. The first two cysteine residues were reacted with xylene dibromide, and the second pair of cysteine residues with Acm protecting groups were deprotected and oxidized by normal iodine oxidation (Figure 2). To increase the yields of the xylene cyclization products, different reactant ratios, concentrations, acetonitrile (ACN)-H_2_O ratios and reaction times were examined on TxIB[1,3]-L. The results showed that the proportion of ACN should be higher than 60%; otherwise, the xylene dibromide cannot dissolve completely. The reactant concentration should not be more than 0.1 mM; otherwise, dialkylated monobromoxylene byproduct p3 was generated (Supplementary Material Figure S2). It can be seen that when the equivalent ratio of peptide to dibromide was 1:5 or 1:10 (Figure 3), the reaction was basically complete within 20 min. Even with this high ratio, little byproduct was generated. In pursuit of an atom-economic reaction, two equivalents of xylene dibromides were used to drive the reaction to completion. Similar conversion rates of TxIB[1,3]-L reacted with m-xylene dibromide and o-xylene dibromide were observed (Supplementary Material Figure S3). Initially, a small amount of disulfide bond byproduct p1 was observed (Supplementary Material Figure S3). This is because, under alkaline conditions, two cysteine residues were oxidized by oxygen to form a disulfide bond. This situation can be avoided under nitrogen protection. Overall, the optimal reaction conditions should be the linear peptide (0.1 mM) and xylene dibromide (0.2 mM) in a cosolvent of 5% volume NH_4_HCO_3_ buffer (0.2 M, pH 8.0), 60% volume ACN and 35% volume H_2_O under nitrogen at room temperature. The reaction normally completes within an hour and the yield is quantitative.

**Figure 4:**
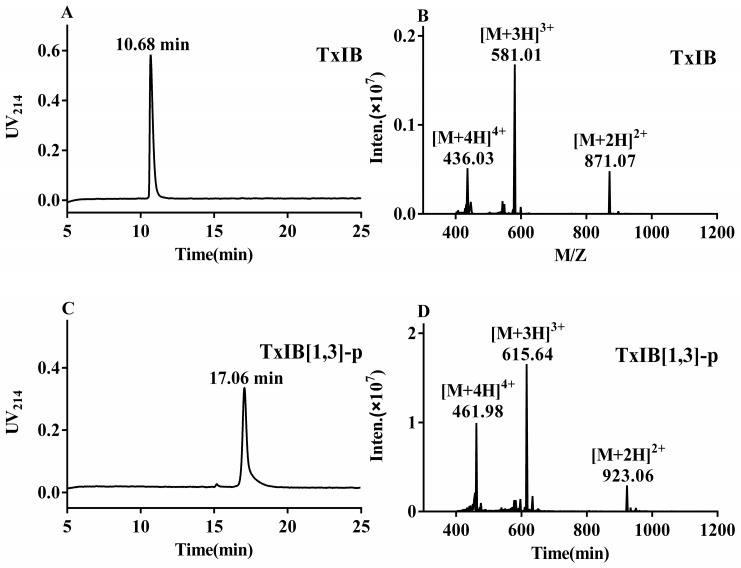
HPLC and mass spectrometry results of TxIB[1,3]-L reacted with p-xylene dibromide. (**A**) HPLC chromatogram of native TxIB with a retention time of 10.68 min. (**B**) ESI-MS data of native TxIB with a mass of 1739.92 Da. (**C**) HPLC chromatogram of TxIB[1,3]-p with the retention time of 17.06 min. (**D**) ESI-MS data of TxIB[1,3]-p with a mass of 1844.12 Da.

With this method, three isomeric xylene dibromides were individually reacted with one pair of the cysteine residues ([1,3] or [2,4]) in the linear peptide, and iodine oxidation was used to form a disulfide bond between the other pair. Finally, all the analogs were synthesized in two steps with isolated yield from 55% to 65%. The purity of TxIB and six analogs were determined by HPLC, and the molecular weights of these peptides were detected by ESI-MS (Figure 4 and Supplementary Material Figures S4 and S5).

### 2.2. Electrophysiological Activity Measurements

To evaluate the activity and selectivity of TxIB after modification, the inhibitory activity of TxIB and its six analogs were tested on α6/α3β2β3 nAChR expressed in *Xenopus* oocytes using electrophysiological assays. The purity of all peptides examined for biological activity was above 95%.

**Figure 5:**
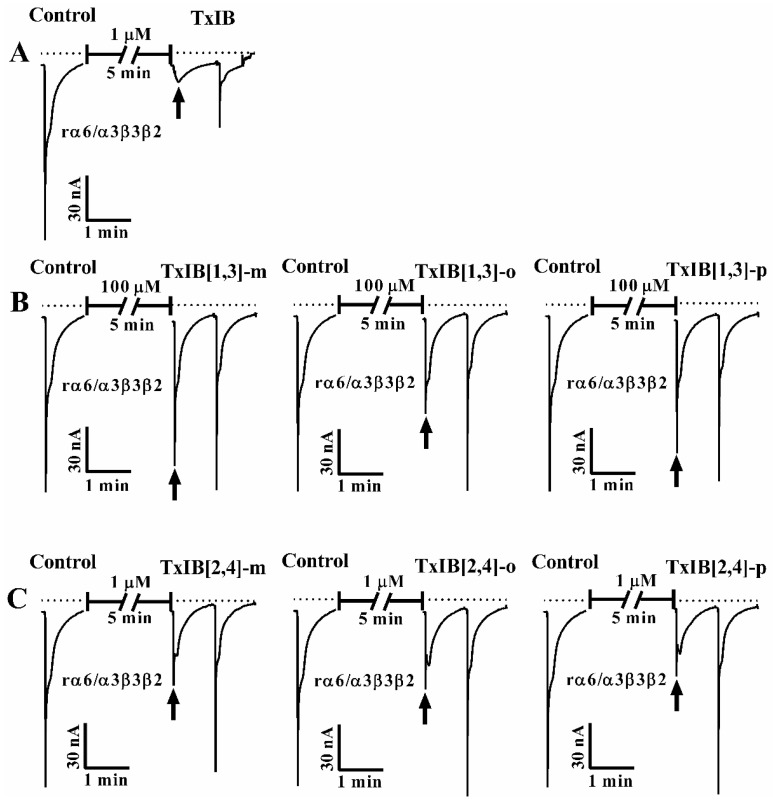
The relative membrane currents of TxIB and its analogs at a concentration of 1 μM and 100 μM on rat α6/α3β2β3 nAChR. The negative control was tested by ND-96 solution only. (**A**) TxIB at 1 μM. (**B**) TxIB[1,3]-m at 100 μM. (**C**) TxIB[2,4]-m at 1 μM.

The preliminary test results showed that cysteine [2,4] modified products have obvious inhibitory activity at 1 μM and cysteine [1,3] modified products showed negligible activity even at 10 μM. 100 μM was selected as the concentration for TxIB[1,3]-m, o, p in the electrophysiological assay. As shown in Figure 5, 1 μM TxIB and TxIB[2,4]-m, o, p showed about 88% and 50%–55% inhibition, respectively, on α6/α3β2β3 nAChR, while the three TxIB[1,3]-m, o, p products only have about 10%–40% inhibition with 100 μM (Supplementary Material Figure S6). As pharmacological activity retention products, TxIB[2,4]-m, o, p were also tested on Mα1β1δε, α3β2, α3β4, α4β2, α6/α3β4, α6/α3β3β2 and α9α10 nAChR subtypes at a concentration of 10 μM (Supplementary Material Figure S7). No significant inhibition was observed, which means the selectivity was maintained. 

### 2.3. Serum Stability of Native Peptide and Its Analogs

**Figure 6:**
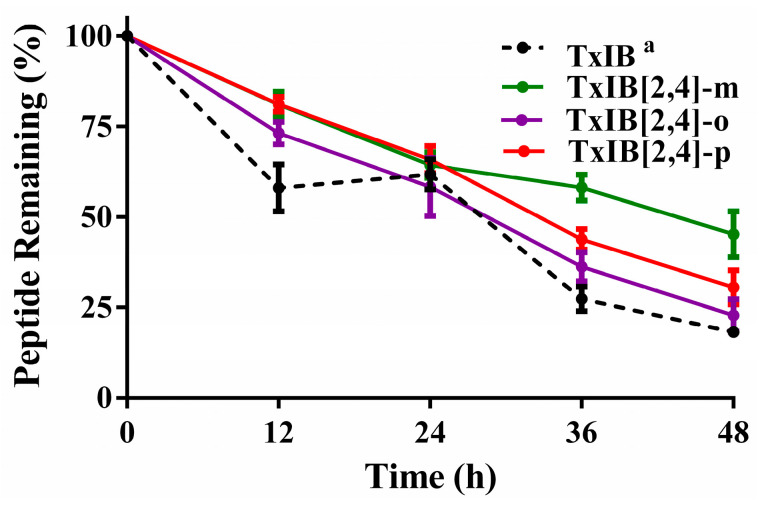
The relative stability of native peptide and the analogs in human serum. Error bars represent the mean ± SEM (*n* = 3), ^a^ taken from ref. [15].

The peptides were incubated in human serum at 37 °C to test their stability. As shown in Figure 6, the stability of the analogs was improved compared with the native TxIB. Among the three active analogs, TxIB[2,4]-m is the most stable one, followed by the TxIB[2,4]-p and TxIB[2,4]-o. The results indicated that the modification on the disulfide bond increased the serum metabolic stability slightly, as (45.2 ± 6.3)% TxIB[2,4]-m remained after 48 h. Based on these data, the circulating half-lives of TxIB and the xylene analogs may be governed by renal and tissue clearance, rather than their intrinsic stability in the circulation.

### 2.4. CD Spectroscopy Assays

**Figure 7:**
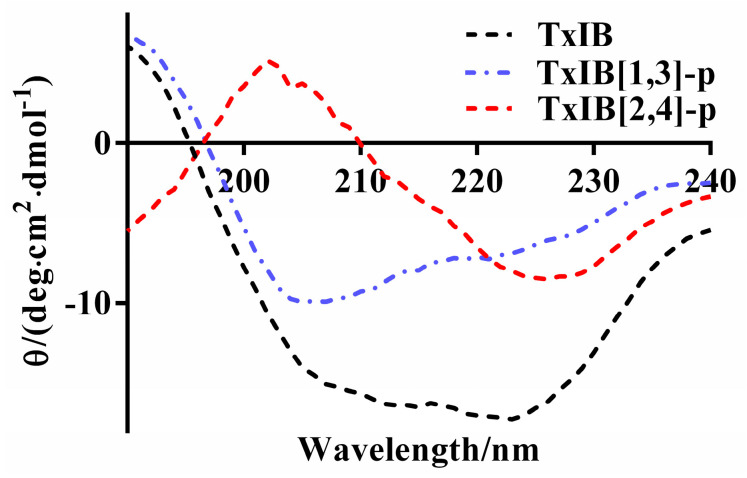
Circular dichroism (CD) spectra of TxIB[1,3]-p and TxIB[2,4]-p compared with native globular TxIB.

CD spectroscopy was used to study the conformational changes between native TxIB and its analogs TxIB[1,3]-p and TxIB[2,4]-p. The absorption spectra of TxIB[1,3]-p and TxIB[2,4]-p were different from native TxIB. The negative ellipticities at 208 nm (λ) and 222 nm (λ) of TxIB showed the existence of α-helix. Meanwhile, the positive Cotton effect and negative Cotton effect at 205 nm of TxIB[1,3]-p and TxIB[2,4]-p indicated the different types of β-turn (Figure 7). 

The secondary structure content was calculated by the Contin-LL program (Provencher & Glockner Method) [33]. Compared to the native TxIB, both analogs have a lower content of α-helix but a higher content of β-sheets (Table 1). We can conclude that the insertion of xylene linkers between cysteine 2 and 4 shaped its new secondary structure.

## 3. Discussion

**Figure 8:**
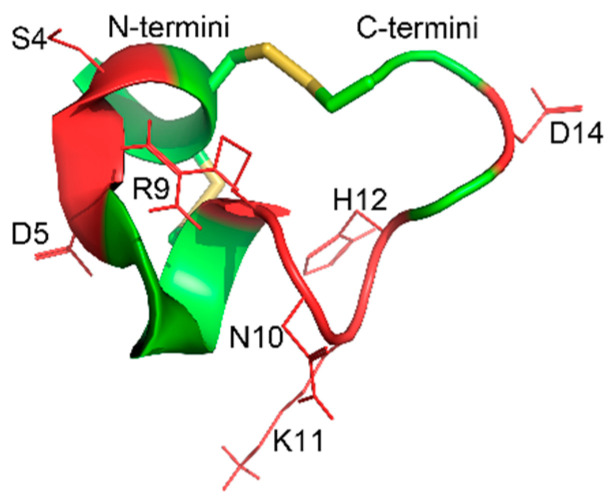
Structure of the globular α-Conotoxin TxIB (PDB identifier 2LZ5).

Most of the previous work on disulfide bond modifications are disulfide bond substitutions. These methods can better maintain the active conformation of polypeptides. Moreover, the introduction of unnatural amino acids and catalysts does not have advantages in synthetic cost and technical difficulty [23]. TxIB contains almost all amino acid residues with reactive side chains, like Ser, Asp, Arg, Asn, Lys and His (Figure 8), which makes selective chemical modification a challenge. This study is based on the direct modification of natural linear peptides with cysteine residues. Specifically, three kinds of xylene (o-, m- and p-) were inserted into the disulfide bond at cysteine [1,3] or cysteine [2,4] one at a time to obtain six peptidomimetics. The experimental results showed that the reaction can be scaled up smoothly and obtain the folded peptides with much higher isolated yields (55% to 65%) than are otherwise currently available. In other words, the yield was increased by four times compared to the traditional method. In general, the method is simple and feasible with low cost and high yield.

Disulfide bonds are generally considered to be essential for pharmacophoric conformation of polypeptides. Although there are many works on conotoxin disulfide bond modification, to the best of our knowledge, all of them are on disulfide bond substitutions. Even a disulfide bond replacement may lead to direct inactivation of the polypeptide. In both studies of [1,3] disulfide bond replacement of α-Conotoxin GI by 1,2,3-triazole and α-Conotoxin RgIA by ethylenic bond, there was biological activity loss against nAChRs [25,34]. It is widely believed that insert modification between the disulfide bond can easily destroy pharmacological activity. Interestingly, our experiments showed that maintaining the TxIB [1,3] disulfide bond and inserting a linker between [2,4] cysteine did not collapse the activity. Compared with TxIB[1,3]-m, o, p, TxIB [2,4]-m, o, p showed activity that was more than 100 times higher. Looking at the three-dimensional structure of TxIB (Figure 8), it is easy to see that the [1,3] disulfide bond in the α-helix region is more important to maintain its spatial structure. Previous studies have shown that the α-helix of conotoxins determines their activities; therefore, we hypothesized that the modified products with retained activity should have higher α-helix content than the modified products with lost activity. Again, surprisingly, the CD spectra showed that the active retained products had the lowest content of α-helix (Table 1). All these findings make us eager to explore further the relationship between structure and activity of this kind of α-Conotoxin. Further disulfide bond insert modifications on different α-Conotoxins with different linkers are under investigation. 

In conclusion, we have found the cysteine [2,4] disulfide bond as a new modifiable site of α-Conotoxin TxIB. This site can be efficiently modified as a bridge connected to useful modules, like drugs and fluorescent molecules. This will provide a basis for the development and utilization of α-Conotoxin TxIB analogs as therapeutic and molecular-probe candidates.

## 4. Materials and Methods 

### 4.1. Reagents and Materials

Acetonitrile (ACN, chromatographically pure grade) was purchased from Fisher Scientific (Pittsburgh, PA, USA); human serum was purchased from Sigma (St. Louis, MO, USA); trifluoroacetic acid (TFA) was purchased from Tedia Company (Fairfield, OH, USA); and m-xylene dibromide, o-xylene dibromide and p-xylene dibromide were purchased from Aladdin (Shanghai, China). All the other reagents used for peptide chemical synthesis were obtained from GL Biochem (Shanghai, China) and Applied Biosystem (Foster City, CA, USA). All of the reagents unless prompted were analytical grade and obtained commercially. HPLC analytical C18 Vydac column (5 µm, 4.6 mm × 250 mm) and preparative C18 Vydac column (10–15 µm, 22 mm × 250 mm) were obtained from HICHROM (UK). ACQUITY UPLC BEH C18 Column (1.7 µm, 2.1 mm × 50 mm) was obtained from Waters (Milford, MA, USA).

### 4.2. Peptide Synthesis

The sequence of linear conotoxin TxIB is GCCSDPPCRNKHPDLC# (# signify C-term amidation). The linear peptides were synthesized on MBHA resin through standard Fmoc solid-phase synthesis and cleaved from the resin by treatment with the cleavage mixtures (TFA-water-phenol-thioanisole-EDT, 82.5:5:5:5:2.5, v:v:v:v:v) for 1.5–2 h. The first and third cysteine residues of TxIB[2,4]-L were protected by Acm, and the second and fourth cysteine residues of TxIB[1,3]-L were protected by Acm. The crude-precipitated peptide was washed with ether (−20 °C) and recycled by centrifuge at 15,000 g for 10 min at 4 °C three times.

### 4.3. General Method for the Synthesis of TxIB[1,3]-m,o,p and TxIB[2,4]-m,o,p

NH_4_HCO_3_ buffer (0.2 M, pH 8.0) was added to a solution of the linear peptide (1 equivalent) in ACN-H_2_O. The solution was mixed by vortexing for 20 s and added to the xylene bromide (2 equivalents) in ACN in one pot. The reaction mixture was mixed by vortexing for another 30 s, and the reaction was carried out on an IKA Loopster Digital (50 rpm) at room temperature. The final composition of the solvents was ACN-H_2_O-NH_4_HCO_3_ buffer (60:35:5, v:v:v), and the final concentration of linear peptide was 0.1 mM [32]. After finishing the reaction, trifluoroacetic acid (0.1% in water) was added to terminate the reaction, the product was separated by preparative HPLC and the product fraction was used directly for the next step by iodine oxidation (0.05 mM peptide, 0.8 mM I_2_, ACN-TFA-water, 30:2:68, v:v:v) for 5–10 min. Saturated vitamin C solution was added to quench the reaction. The target product was separated and purified by preparative HPLC with a reversed-phase C18 Vydac column. The chromatographic conditions were 5%–40% buffer B with a flow rate of 10 mL/min at UV-214 nm within 35 min, and the column temperature was 40 °C. The buffer A was 0.1% TFA in H_2_O, and buffer B was 0.1% TFA in ACN. The UPLC-MS was used to characterize the products. 

### 4.4. Electrophysiological Activity on Different nAChRs

The samples were dissolved in ND-96 solution (96.0 mM NaCl, 1.0 mM MgCl_2_, 2.0 mM KCl, 1.8 mM CaCl_2_, 5mM HEPES, pH 7.1–7.5) for use. The plasmids of mouse (α1, β1, δ and ε) and rat (α3, α4, α6/α3, α7, α9, α10, β2, β3 and β4) nAChR subunits were linearized by corresponding enzymes for in vitro cRNA transcription using the mMessage mMachine kit (Ambion, Austin, TX, USA). The cRNA was purified by the MEGA Clear Kit (Ambion). The cRNA of different nAChR subunits were combined in a specific ratio and injected into *Xenopus* oocytes (within 59.6 nL). The cRNA-injected oocytes were incubated at 17 °C in ND-96 buffer with an antibiotic (10 mg/L penicillin, 10 mg/L streptomycin and 100 mg/L gentamicin) for 1–5 days. Then the oocyte was fixed in the oocyte chamber (a cylindrical well, 50 µL in volume) and ND-96 solution (1 µM atropine and 0.1 mg/mL bovine serum albumin) was gravity-perfused at a rate of 2 mL/min. The membrane currents of the *Xenopus* oocytes expressed different subtypes of nAChR and were recorded by a two-electrode voltage clamp. The membrane potential of the oocytes was clamped at −70 mV. Two seconds of 100 µM ACh and another 58 seconds of ND-96 were applied to the oocyte, which was repeated until a stable baseline was obtained three times. Next, the oocytes were incubated in either ND-96 or different concentrations of conotoxins in ND-96 for 5 min, followed by Ach stimulation (2 s 100 µM Ach and 58 s ND-96) repeatedly. The membrane currents were recorded by a two-electrode voltage clamp at room temperature. All the nAChR inhibitory activities were tested in this method.

### 4.5. Stability Assays 

Male AB human serum was employed for the serum stability test. The serum was pretreated by centrifuge (15,000 g, 15 min) to separate the lipids, and the supernatant was taken out and incubated at 37 °C for 10 min before use. The peptide samples were dissolved in serum at a concentration of 0.1 mM and incubated in a 37 °C constant temperature incubator. Then, the aliquots of each peptide were taken out at 0, 12, 24, 36 and 48 h, quenched by an equal volume of urea (6 M in H_2_O) and incubated for 10 min at 4 °C. An equal volume of 20% TFA was used to precipitate proteins for 10 min at 4 °C. All aliquots were centrifuged at 15,000 g for 10 min, and the supernatant was analyzed by HPLC using an elution gradient of 10–50% buffer B for 25 min. The remaining amounts of peptide were detected and quantified by the peak area at UV-214 nm. All the experiments were repeated at least three times.

### 4.6. Circular Dichroism Spectroscopy

Jasco J-810 spectropolarimeter was employed to test the CD spectra of native TxIB and its analogs with 10 mm path length quartz cuvette. The concentration of peptides was 0.1 mg/mL in water. The spectra were measured in the far UV region (190–240 nm) using an average of 10 scans. The experimental parameters of scanning speed were 100 nm/min, 0.5 s response time, 100 millidegrees sensitivity range and 1 nm step resolution to keep stable measurement conditions, the flow of nitrogen was maintained at 15 mL/min and all experiments were carried out at room temperature (26 °C). The data was analyzed and processed by DichroWeb [35].

## Figures and Tables

**Table 1:** Secondary structure contents of TxIB and its analogs.

Name	Secondary Structures			
	α-Helix	β-Sheet	β-Turns	Random Coil
TxIB	38.2%	6.4%	22.1%	33.3%
TxIB[1,3]-p	23.3%	21.7%	23.3%	31.7%
TxIB[2,4]-p	18.8%	27.2%	20.7%	33.3%

## Data Availability

All data is contained within this article and supplementary materials.

## Supplementary Materials

The following are available online at https://www.mdpi.com/1660-3397/19/2/119/s1, Figure S1: HPLC chromatogram and mass spectra of the linear peptides TxIB[1,3] and TxIB[2,4]. Figure S2: HPLC chromatogram and MS spectra of the linear peptide TxIB[1,3] reaction with p-xylene dibromide. Figure S3: A. The conversion rate of TxIB[1,3] with m-xylene dibromide. B. The conversion rate of TxIB[1,3] with o-xylene dibromide. Figure S4: HPLC chromatogram and MS spectra of TxIB[1,3]-m and TxIB[1,3]-o. Figure S5: HPLC chromatogram and MS spectra of TxIB[2,4]-m, o, p. Figure S6: The inhibition of TxIB and its analogs on nAChR subtypes expressed in *Xenopus laevis* oocytes. Figure S7: The response histogram of TxIB[1,3]-m, o, p on Mα1β1δε, α3β2, α3β4, α4β2, α6/α3β4 and α9α10 nAChR subtypes.
