# Supplementary material for: Cysteine [2,4] Disulfide Bond as a New Modifiable Site of α-Conotoxin TxIB
Source: Mar Drugs. 2021 Feb 22;19(2):119. doi: 10.3390/md19020119 (PMC7926623; doi:10.3390/md19020119)
Supplement: Supplementary file 1 [file marinedrugs-19-00119-s001.pdf]

Supporting Information

Cysteine [2,4] Disulfide Bond as a New Modifiable Site of α-Conotoxin TxIB

Baojian Zhang [1], Maomao Ren [1], Yang Xiong [1], Haonan Li [1], Yong Wu [2], Ying Fu [1], Dongting Zhangsun [1,2], Shuai Dong [1*] and Sulan Luo [1,2*]

[1] Key Laboratory of Tropical Biological Resources of Ministry of Education, Key Laboratory for Marine Drugs of Haikou, School of Life and Pharmaceutical Sciences, Hainan University, Haikou 570228, China; zhangbaojian96@163.com(B.Z.); syphurmm@163.com (M.R.); ncdxxy@163.com (Y.X.); lihaonan_suger@163.com (H.L.); fuying926@163.com (Y.F.); zhangsundt@163.com (D.Z.)

[2] Medical School, Guangxi University, Nanning 530004, China; wys211@163.com (Y.W.)

* Correspondence: dongshuai_1024@163.com (S.D.); luosulan2003@163.com (S.L.)

Tel.: +86-898-6628-9538(S.D. & S.L.)

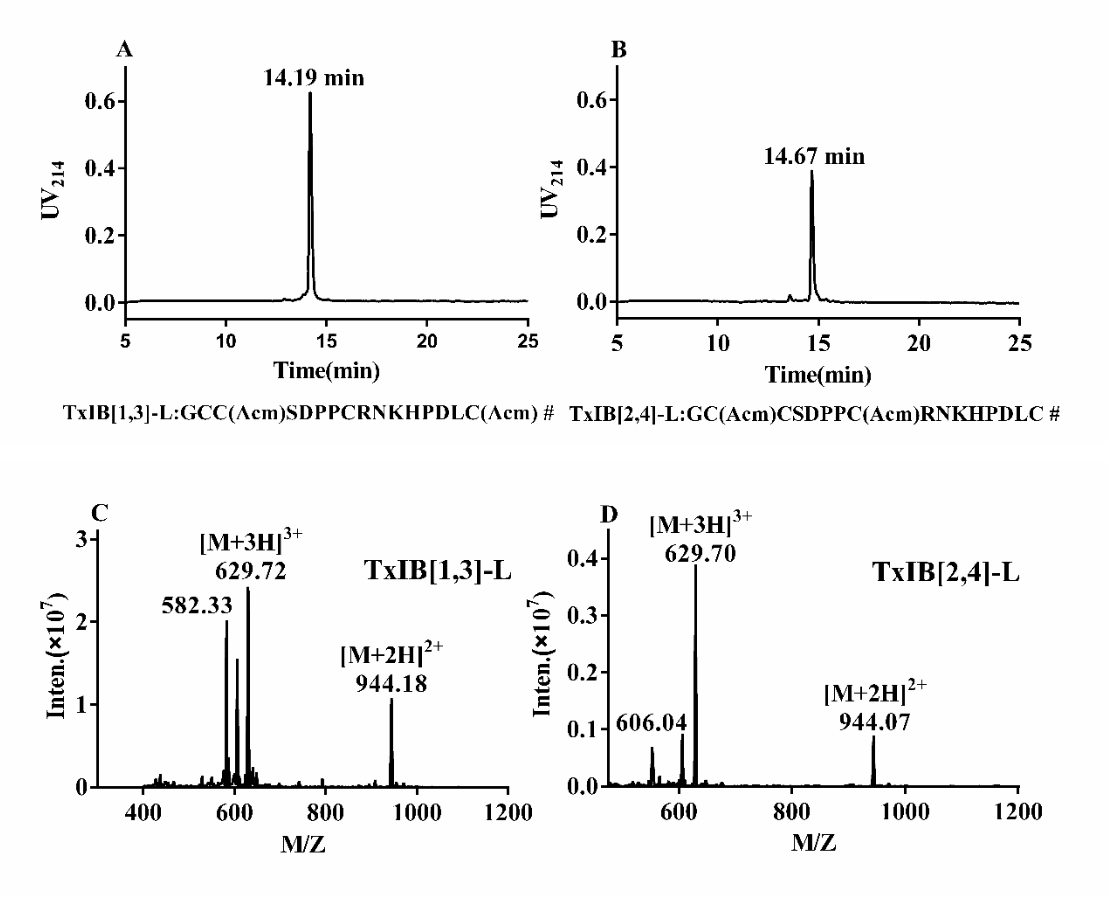

Figure S1. HPLC chromatogram and mass spectra of the linear peptides TxIB[1,3] and TxIB[2,4].

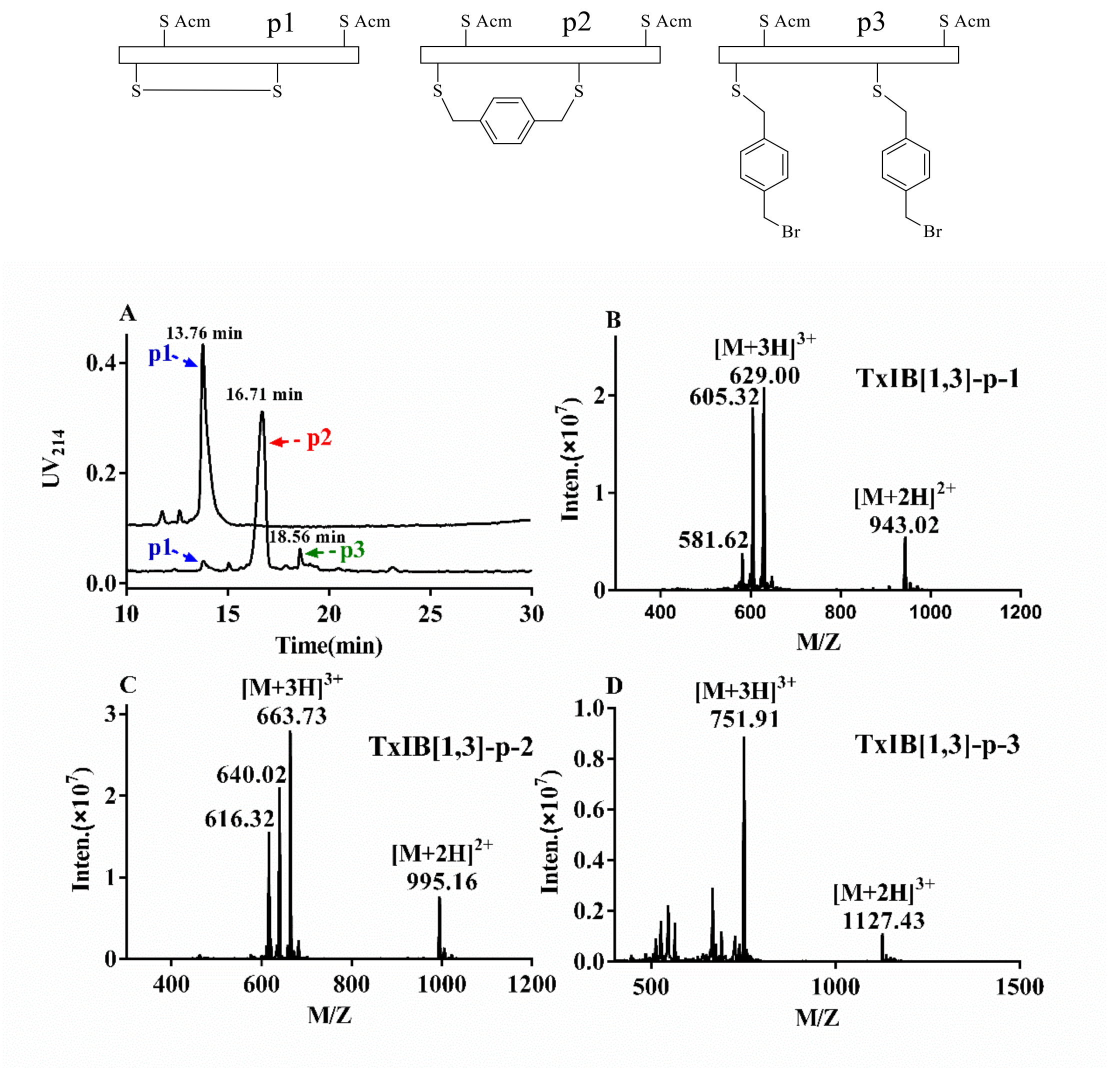

Figure S2. HPLC chromatogram and MS spectra of the linear peptide TxIB[1,3] reaction with p-xylene dibromide.

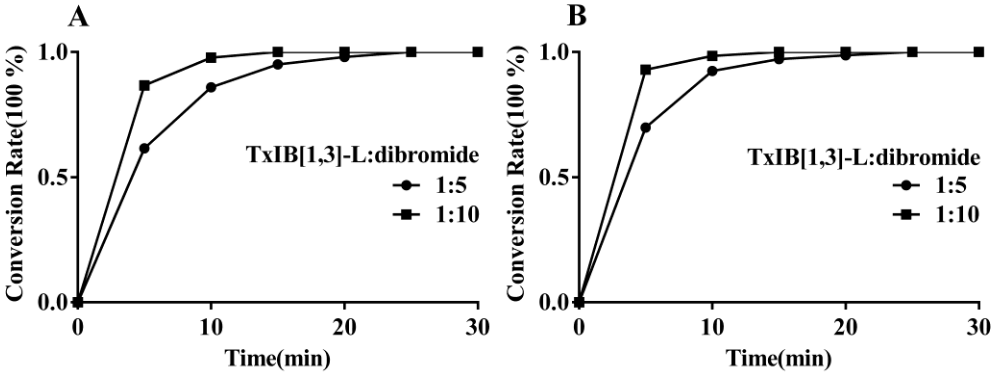

Figure S3. A. The conversion rate of TxIB[1,3]-L with m-xylene dibromide. B. The conversion rate of TxIB[1,3]-L with o-xylene dibromide.

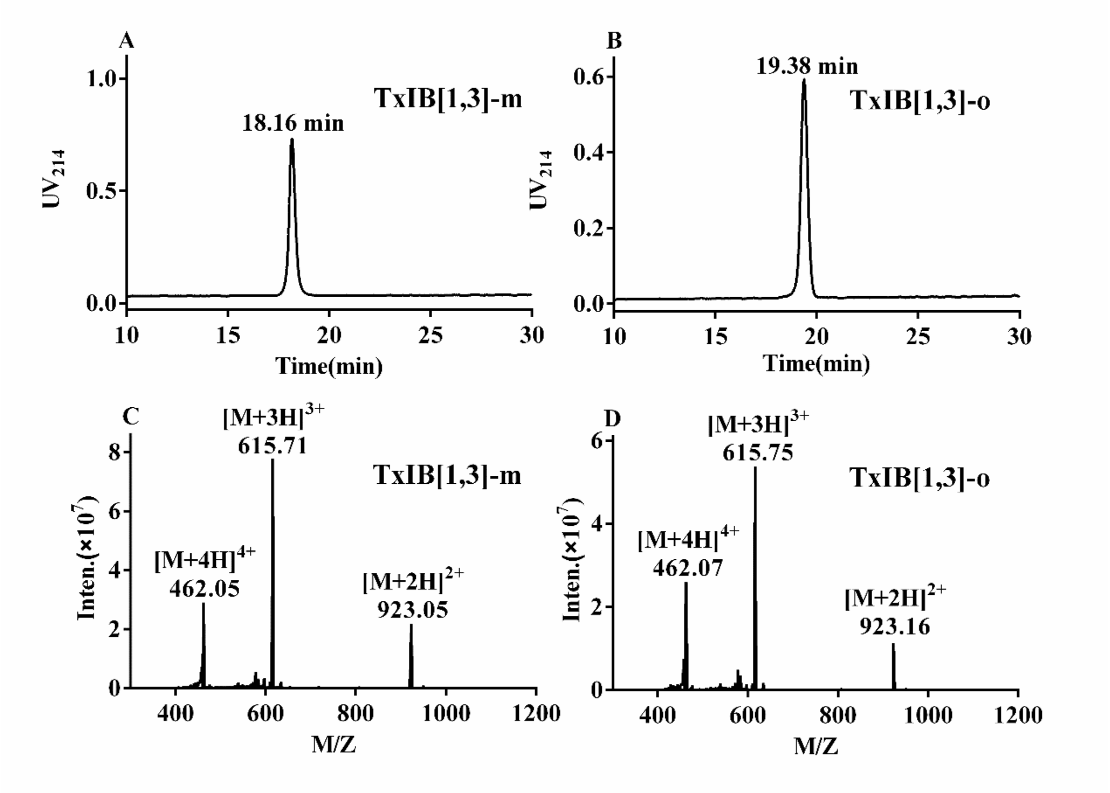

Figure S4. HPLC chromatogram and MS spectra of TxIB[1,3]-m and TxIB[1,3]-o.

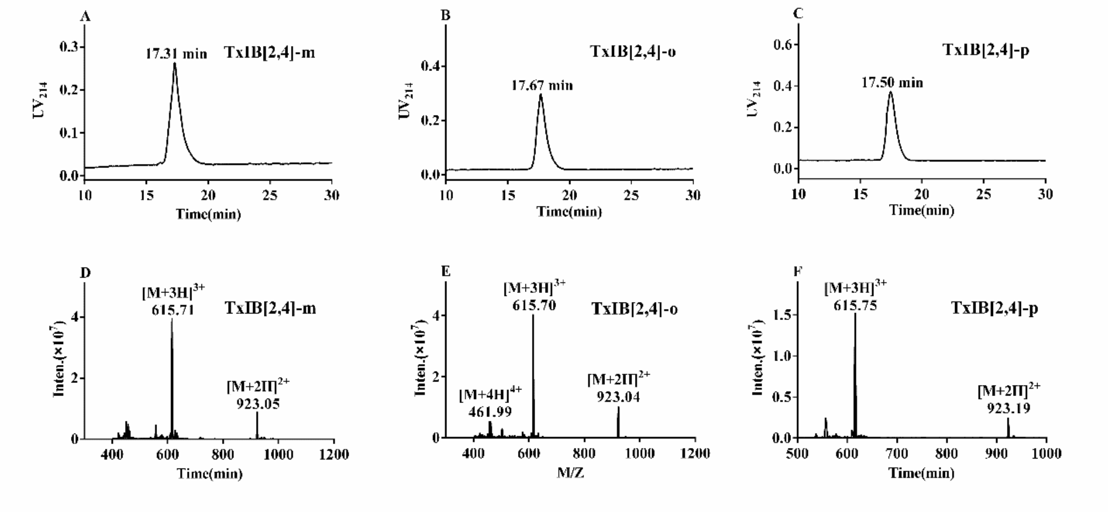

Figure S5. HPLC chromatogram and MS spectra of TxIB[2,4]-m, o, p.

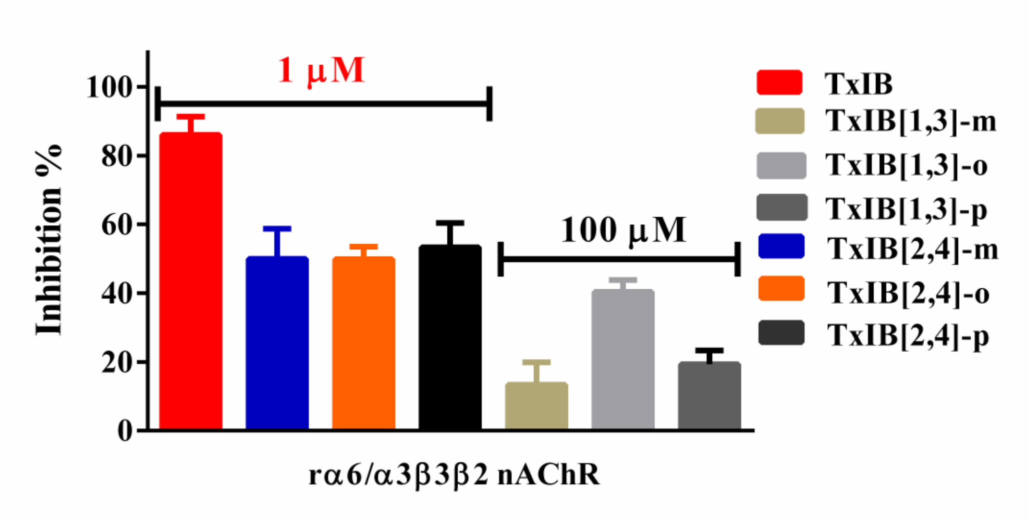

Figure S6. The inhibition of TxIB and its analogs on nAChR subtypes expressed in *Xenopus laevis* oocytes. TxIB and [2,4] modified analogs were determined at the concentration of 1 μM and [1,3] modified products were determined at the concentration of 100 μM on α6/α3β3β2 nAChR. All data represent mean ± S.E.M, n = 3–6.

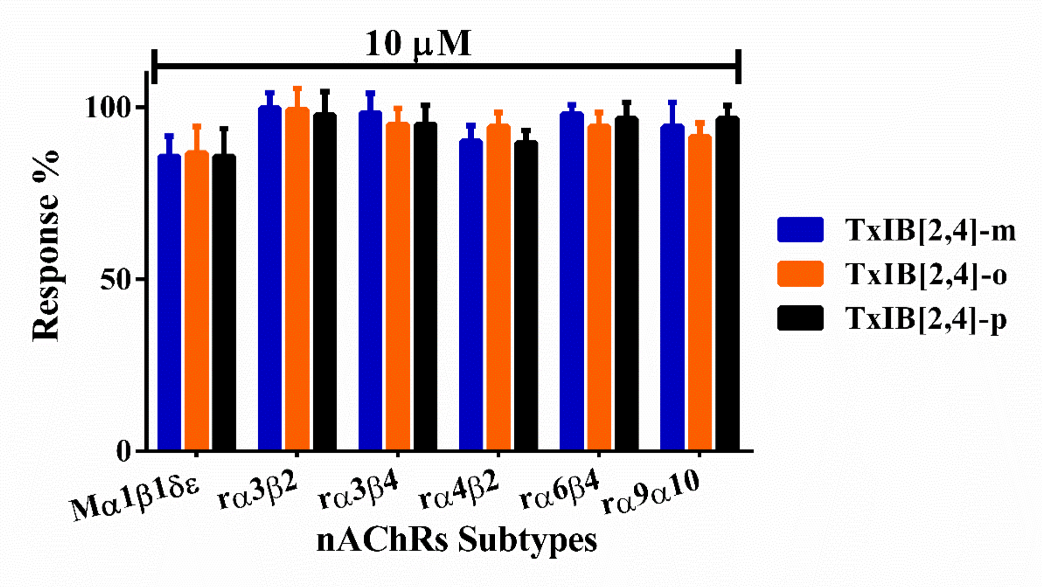

Figure S7. The response histogram of TxIB[1,3]-m, o, p on Mα1β1δε, α3β2, α3β4, α4β2, α6/α3β4 and α9α10 nAChR subtypes. All data represent mean ± S.E.M, n = 3–6.
